# Antidiabetic Biguanides Radiosensitize Hypoxic Colorectal Cancer Cells Through a Decrease in Oxygen Consumption

**DOI:** 10.3389/fphar.2018.01073

**Published:** 2018-10-03

**Authors:** Sven de Mey, Heng Jiang, Cyril Corbet, Hui Wang, Inès Dufait, Kalun Law, Estelle Bastien, Valeri Verovski, Thierry Gevaert, Olivier Feron, Mark De Ridder

**Affiliations:** ^1^Department of Radiotherapy, Universitair Ziekenhuis Brussel, Vrije Universiteit Brussel, Brussels, Belgium; ^2^Pole of Pharmacology and Therapeutics, Institut de Recherche Expérimentale et Clinique (IREC), Université catholique de Louvain, Brussels, Belgium; ^3^Laboratory of Molecular and Cellular Therapy, Vrije Universiteit Brussel, Brussels, Belgium

**Keywords:** phenformin, metformin, hypoxic radiosensitivity, mitochondrial complex I, oxygen consumption rate, colorectal cancer

## Abstract

**Background and Purpose:** The anti-diabetic biguanide drugs metformin and phenformin exhibit antitumor activity in various models. However, their radiomodulatory effect under hypoxic conditions, particularly for phenformin, is largely unknown. This study therefore examines whether metformin and phenformin as mitochondrial complex I blockades could overcome hypoxic radioresistance through inhibition of oxygen consumption.

**Materials and Methods:** A panel of colorectal cancer cells (HCT116, DLD-1, HT29, SW480, and CT26) was exposed to metformin or phenformin for 16 h at indicated concentrations. Afterward, cell viability was measured by MTT and colony formation assays. Apoptosis and reactive oxygen species (ROS) were detected by flow cytometry. Phosphorylation of AMP-activated protein kinase (AMPK) was examined by western blot. Mitochondria complexes activity and oxygen consumption rate (OCR) were measured by seahorse analyzer. The radiosensitivity of tumor cells was assessed by colony formation assay under aerobic and hypoxic conditions. The *in vitro* findings were further validated in colorectal CT26 tumor model.

**Results:** Metformin and phenformin inhibited mitochondrial complex I activity and subsequently reduced OCR in a dose-dependent manner starting at 3 mM and 30 μM, respectively. As a result, the hypoxic radioresistance of tumor cells was counteracted by metformin and phenformin with an enhancement ratio about 2 at 9 mM and 100 μM, respectively. Regarding intrinsic radioresistance, both of them did not exhibit any effect although there was an increase of phosphorylation of AMPK and ROS production. In tumor-bearing mice, metformin or phenformin alone did not show any anti-tumor effect. While in combination with radiation, both of them substantially delayed tumor growth and enhanced radioresponse, respectively, by 1.3 and 1.5-fold.

**Conclusion:** Our results demonstrate that metformin and phenformin overcome hypoxic radioresistance through inhibition of mitochondrial respiration, and provide a rationale to explore metformin and phenformin as hypoxic radiosensitizers.

## Introduction

Metformin, an oral biguanide, is the most widely used drug to treat type II diabetes. Epidemiological evidence indicates that metformin reduces cancer risk and enhances response to therapeutics, consequently decreasing cancer-related mortality (Decensi et al., 2010; Quinn et al., 2013). Currently, repurposing metformin as an anti-cancer drug is receiving considerable attention with over 100 clinical trials, wherein its anti-cancer effect is explored alone or combined with other therapies (Chae et al., 2016). The enthusiasm toward repurposing metformin is extended to another biguanide anti-diabetic drug, namely phenformin. As a therapeutic for diabetes, its use has been limited to relatively few countries due to an increased incidence of lactic acidosis in elderly patients with renal failure (0.4–0.64 cases per 1,000 patient) (Pernicova and Korbonits, 2014; Liu et al., 2015). While as a potential anti-cancer drug, phenformin showed much higher cytotoxic activity toward tumor- and tumor-supporting cells compared with metformin in various preclinical models (Shackelford et al., 2013; Gravel et al., 2014; Velez et al., 2016).

The mechanisms by which metformin and phenformin inhibit cancer development and tumor growth are not completely elucidated; whereas emerging evidence suggests that inhibition of mitochondrial complex I is one of the major mechanisms. Firstly, inhibition of complex I by metformin and phenformin is associated with an increased cancer cell susceptibility to apoptosis via changing the topology of the inner mitochondrial membrane (Velez et al., 2016). Secondly, inhibition of complex I increases the aberrant flow of electrons to oxygen and creates reactive oxygen species (ROS), subsequently causing damage to proteins, lipids, and nucleic acids (Miskimins et al., 2014). Consistently, metformin and phenformin eliminate tumor cells and tumor-supporting cells such as myeloid-derived suppressor cells (MDSC) through overproduction of ROS (Haugrud et al., 2014; Miskimins et al., 2014; Kim et al., 2017). Thirdly, inhibition of complex I results in impaired mitochondrial metabolism. Thus, the cytotoxic effect of metformin and phenformin is particularly vigorous in cancer cells with a defect in oxidative phosphorylation or glucose uptake (Shackelford et al., 2013; Momcilovic et al., 2015). Intriguingly, perturbation of metabolism by phenformin is reported to reduce stem cell features, leading to decreased cell viability and invasion (Petrachi et al., 2017). Mitochondrial complex I inhibition is an important action by which metformin and phenformin reduce tumor burden, but other mechanisms underlying the anti-cancer effects should not be neglected. Attributed to the antidiabetic properties, metformin reduces circulating glucose and insulin, resulting in a decrease in cell proliferation and invasion; this is particularly effective in tumor with high expression of insulin receptors, such as breast cancer (Goodwin et al., 2012; Mallik and Chowdhury, 2018). In addition, metformin and phenformin trigger the activation of AMP-activated protein kinase (AMPK) that in turn antagonizes the activity of pro-tumor mammalian target of rapamycin (mTOR) signaling, leading to delayed tumor growth and reduced metastasis (Lin et al., 2015; Orecchioni et al., 2015; Navarro et al., 2016).

Complex I is the gatekeeper of the respiratory chain and its inhibition will subsequently result in a decreased oxygen consumption rate (OCR), which is currently considered as a potent strategy to counteract hypoxic radioresistance due to reoxygenation of hypoxic tumor cells (Jiang et al., 2013; Zannella et al., 2013). Hypoxia is a common feature of the tumor microenvironment and considered to be one of the principle causes of clinical failure of radiotherapy (Gray et al., 1953). complex I inhibitors such as arsenic trioxide exhibited clear radiosensitizing effect in solid tumors via increasing tumor oxygenation (Paul et al., 2008; Diepart et al., 2012). Metformin and phenformin have been both characterized as complex I inhibitors and reported to markedly block cellular respiration in diverse tumor cells (Wheaton et al., 2014; Ashton et al., 2016), while their hypoxic radiosensitizing effect, particularly for phenformin, is still largely unexplored. Regarding their impact on aerobic (intrinsic) radiosensitivity, both of them have been reported to radiosensitize aerobic tumor cells, such as lung and breast cancer cells (Storozhuk et al., 2013; Zhang et al., 2014; Wang et al., 2015a), however, this effect is not yet well defined in colorectal cancer (CRC) – the research focus in our department. In addition, the difference in their radiosensitizing potency is almost unknown, even though their cytotoxic effects have been intensively studied in parallel due to the similarity of chemical structure and the common molecular target. In this study, we therefore examined the radiomodulatory effect of metformin and phenform in a panel of CRC cells and further validated our findings in tumor-bearing mice.

## Materials and Methods

### Cell Lines and Chemicals

Murine CT26 and human HCT116, DLD-1, HT29, and SW480 CRC cell lines were obtained from American Type Culture Collection (ATCC, Manassas, United States). All experiments were performed in RPMI 1640 medium (Thermo Fisher, Belgium) supplemented with 10% bovine calf serum (Greiner Bio-One, Belgium). Chemicals were obtained from Sigma-Aldrich (Antwerp, Belgium) unless otherwise stated.

### Treatments

Cells were grown to subconfluence and exposed to metformin or phenformin (Santa Cruz Biotechnology, Dallas, TX, United States) for 16 h at indicated concentrations. The ROS scavenger N-acetyl cysteine (NAC) was added at 10 mM to cultures both 1 h prior and during treatment with metformin or phenformin. Afterward, cultures were used for further analysis as described below.

### MTT Assay

Cytotoxicity of metformin and phenformin was assessed by MTT assay as described elsewhere (Wang et al., 2017). Briefly, after treatment, medium was aspirated and 50 μl MTT solution (5 mg/ml) was added for 1.5 h. Afterward, 200 μl of MTT solvent (19:1 DMSO: HCL) was added and admixed to dissolve the formazan crystals generated inside of cells. Absorbance was measured at a wavelength of 540 nm by using a spectrophotometer (Bio-Rad, CA, United States). Cell viability was determined by dividing the absorbance values of treated cells to that of untreated (control) cells.

### Apoptotic Assay

Apoptosis was analyzed by flow cytometry using the double staining with lipophilic Annexin V and 7-amino actinomycin D (7-AAD) (Abcam, Cambridge, United Kingdom), as described elsewhere (Wang et al., 2017). Briefly, after treatment, cells were harvested and washed with FACS buffer. Thereafter, cells were resuspended in 100 μl binding buffer (eBioscience) with 2.5 μl Annexin V (eBioscience) and incubated for 20 min at room temperature. 7-AAD (5 μl) was added 5 min before analysis. Early apoptotic cells (Annexin V-positive, 7-AAD-negative), necrotic/late apoptotic cells (double-positive), and living cells (double-negative) were determined by flow cytometry (BD LSR Fortsessa, BD Bioscience, Franklin Lakes, NJ, United States).

### Radiation and Clonogenic Assay

After treatment, cells were either subjected to metabolic hypoxia in a micropellet model (Janssens et al., 1998, 1999) or irradiated in suspension. Briefly, to generate micropellets, 0.5 × 10^6^ cells were collected into a 15 ml conical tube and centrifuged at 2000 rpm with a volume of 100 μl. Then just before radiation, the micropellets were placed in a 37°C water bath for 5 min in order to metabolically consume oxygen and induce hypoxia. For cell suspension, 0.5 × 10^6^ cells were collected into a 15 ml conical tube and suspended into 1 × 10^6^/ml, and then vortexed and placed in a 37°C water bath just before radiation. Afterward, radiation was conducted by using a 6 MV Linac (Elekta, Crowley, United Kingdom) at a rate of 2 Gy/min; cells were reseeded for colony formation, as described elsewhere (Jiang et al., 2010). After 7–12 days, cultures were fixed with crystal violet and colonies (>50 cells) were counted. Survival curves were fitted to the linear quadratic model using GraphPad Prism 6 software (GraphPad Prism Software Inc., La Jolla, CA, United States). Radiosensitization was expressed as an enhancement ratio determined at a survival fractions (SF) of 0.1.

### Mitochondrial Complexes Activity

After treatment, mitochondria were isolated from cancer cells and then seeded into a 96 well plate. Afterward, the activity of mitochondrial complexes (I, II, III, and IV) was determined by Seahorse XF96 analyzer (Agilent, Belgium) as depicted in **Supplementary Figure 1** and described elsewhere (Corbet et al., 2016). Briefly, the mitochondrial complexes activity is reflected by the change of OCR in different phases, following the injection of distinct compounds: 10 mM pyruvate, 5 mM malate, 2 mM ADP, 1 μM rotenone, 10 mM succinate, 4 μM antimycin A, and 10 mM ascorbate plus 0.1 mM TMPD.

### Oxygen Consumption Rates

Oxygen consumption rate was determined by Seahorse XF96 analyzer (Agilent, Belgium). Briefly, 2.0 × 10^5^ cells were seeded in 96-well plates. Twenty-four hours later, OCR of the cells was measured in real-time for 8 h after injection of various concentrations of metformin or phenformin. OCR was normalized to the basal level.

### ROS Production

The intracellular level of ROS was detected by flow cytometry using an oxidation sensitive fluorescent probe 5-(6)-chloromethyl-2′,7′-dichlorodihydro-fluorescein diacetate (CM-H2DCFDA) (Abcam, Cambridge, United Kingdom), as described elsewhere (Wang et al., 2017). Briefly, cells were treated with metformin or phenformin for 16 h, stained with 5 μM (CM-H2DCFDA) at 37°C for 30 min and analyzed by flow cytometry.

### Western Blotting

Western blot was performed as described elsewhere (Jiang et al., 2013). Briefly, cells were lysed by sample buffer (Tris-HCL: 62.5 mM pH 6.8, glycerol: 10%, SDS: 2%, β-mercaptoethanol: 5%, Bromophenol: 0.025%). Proteins were then separated on a 12.5% SDS-polyacrylamide gel (Bio-Rad, Hercules, CA, United States) and transferred to nitro-cellulose membrane. Thereafter, membranes were incubated with primary antibodies against AMPK (cell signaling, 5831), phospho-AMPK (cell signaling 2535), and anti-beta actin (cell signaling, 4970). Later, membranes were washed and incubated with appropriate horseradish peroxidase (HRP)-conjugated secondary antibody. Image was taken using the Odyssey Fc imaging system (LI-COR, Belgium).

### Mouse Tumor Model

CT26 tumor cells (0.5 × 10^6^) were inoculated into the left hind limb of syngeneic Balb/c mice (female, 7–9 weeks old; Charles River Laboratories, L’Arbresle Cedex, France). At day 7, mice were randomized and treated with metformin (300 mg/kg) or phenformin (200 mg/kg) through oral gavage for 10 days. Radiation (9Gy) was delivered by using a 6 MV Linac (Elekta, Crowley, United Kingdom) 1 h after the first administration of metformin or phenformin. The tumor volume was calculated using the formula Volume = (Length^∗^Width^2^)^∗^0.5. Experiments were reviewed by the Ethical Committee for use of laboratory animals of the Vrije Universiteit Brussel.

### Statistics

All analyses were performed using prism 6.01 (GraphPad, La Jolla, CA, United States). One-tailed *t*-test, one-way ANOVA followed by a Dunnett’s multiple comparison test and two-way ANOVA with Bonferroni’s multiple comparison test were performed. Sample sizes and number of repetitions were indicated in the figure legends.

## Results

### Cytotoxicity of Metformin and Phenformin Toward a Panel of CRC Cells

**FIGURE 1:**
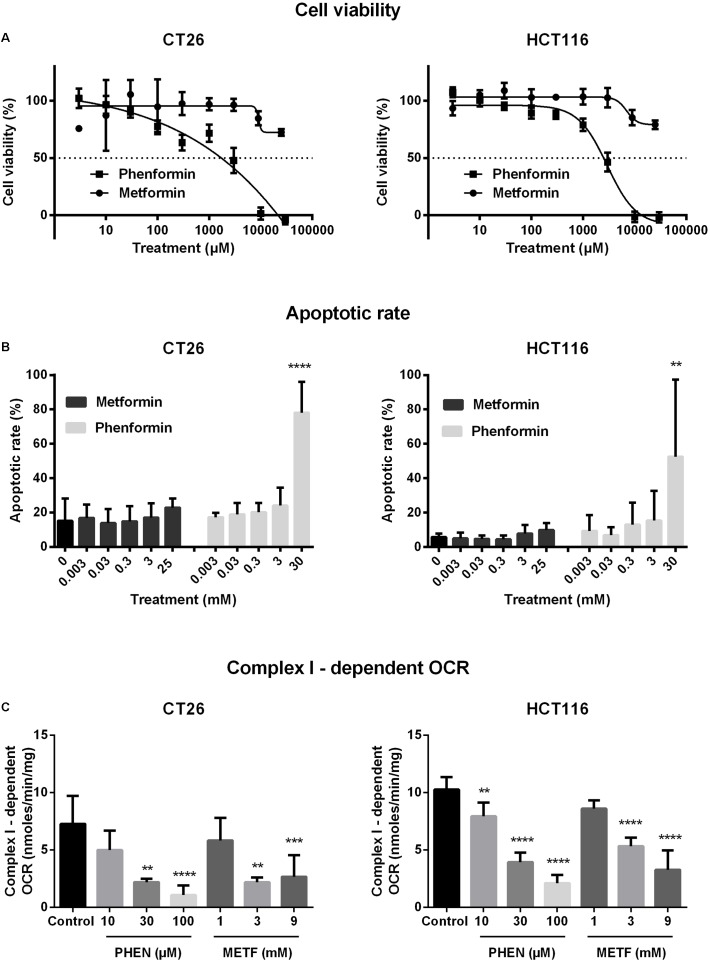
Metformin and phenformin inhibit mitochondrial complex I activity at non-toxic concentrations. CT26 and HCT116 tumor cells were treated with metformin (METF) or phenformin (PHEN) with indicated concentrations for 16 h. Cytotoxicity was determined by using MTT assay **(A)**. Apoptosis was assessed by flow cytometry using Annexin V/7-AAD staining **(B)**. Mitochondria were isolated and activity of mitochondrial complexes I was measured by using a Seahorse analyzer **(C)**. Data are shown from at least three replicates as mean ± SEM. A one-way ANOVA with Dunnett’s multiple comparison test was used to calculate statistics: ^∗^*p* < 0.05, ^∗∗^*p* < 0.01, ^∗∗∗^*p* < 0.001, ^∗∗∗∗^*p* < 0.0001.

The cytotoxic properties of metformin and phenformin were determined by MTT in a panel of CRC cells. Phenformin reduced cell viability in a dose-dependent manner with IC50 values of 2.9–5.2 mM, wherein SW480 is the most sensitive followed by HCT116, CT26, DLD-1, and HT29 (**Figure 1A** and **Supplementary Figure 2A**). At high concentrations (>3mM), a strong induction of apoptosis, mainly late phase, was detected by flow cytometry (**Figure 1B** and **Supplementary Figure 2B**). Oppositely, no considerable effect of metformin on cell viability was observed at doses up to 10 mM (**Figures 1A,B** and **Supplementary Figures 2A,B**). At this point, all four human CRC cell lines demonstrated similar cytotoxic profiles after exposure to metformin and phenformin. For practical reasons the subsequent experiments were limited to one murine and one human cell line: namely CT26 and HCT116.

### Metformin and Phenformin Inhibited Complex I Activity and Impaired Oxygen Consumption

**FIGURE 2:**
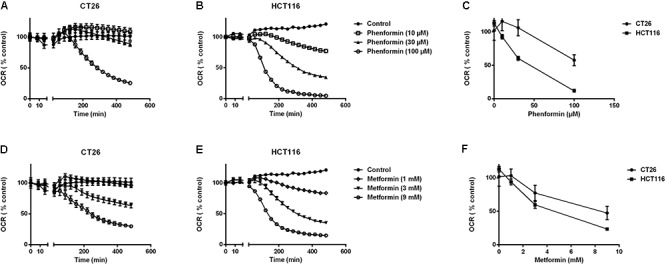
Metformin and phenformin inhibit oxygen consumption. The oxygen consumption rate (OCR) of CT26 **(A,D)** and HCT116 **(B,E)** was measured over time after injection of indicated concentrations of metformin or phenformin using the Seahorse analyzer. The OCR was expressed as a percentage relative to control. Relative OCR compared across the two cell lines at 3 h post-phenformin **(C)** or metformin **(F)** injection. Data is shown as mean ± SEM.

At non-toxic doses, we then evaluated the effect of metformin and phenformin on mitochondrial complexes activity by using a Seahorse analyzer. Phenformin inhibited complex I activity with a dose starting at 10 μM and reaching a more than 50% inhibition at 100 μM in both CT26 and HCT116 (**Figure 1C**). Mitochondrial complex II, III and IV were unaffected by phenformin in both cell lines (**Supplementary Figures 3A–C**). Consequently, phenformin caused a time-dependent decrease in OCR in CT26 at a dose of 100 μM (**Figure 2A**). Compared with CT26, HCT116 is more sensitive to phenformin with a significant inhibition of OCR at a dose of 30 μM (**Figure 2B**). The difference between these two cell lines in response to phenformin was further summarized in **Figure 2C**. Similarly, metformin specifically inhibited the activity of complex I and thereby time and dose dependently decreased cellular respiration in both CT26 and HCT116 (**Figures 1C**, **2D–F**). However, this effect of metformin required concentrations >1 mM, indicating that phenformin is much more potent than metformin to modulate mitochondrial activity.

### Metformin and Phenformin Radiosensitized Hypoxic Tumor Cells

**FIGURE 3:**
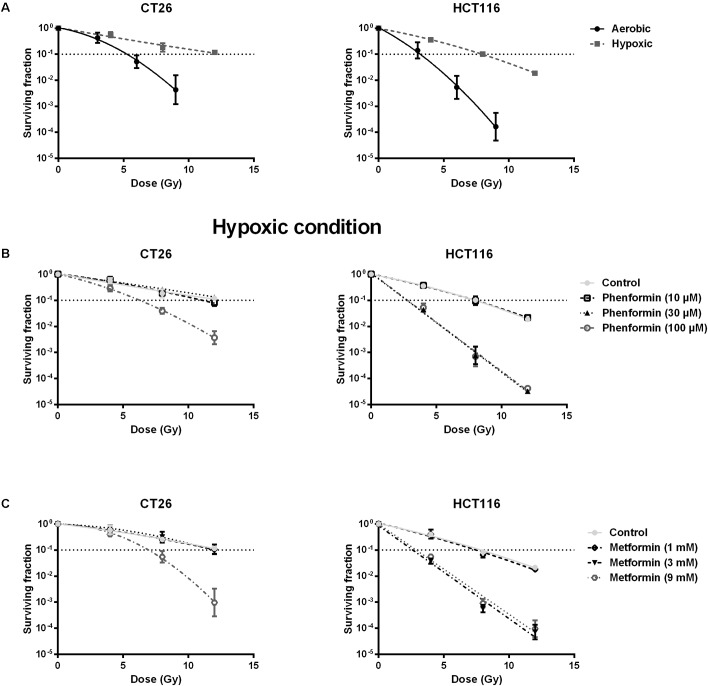
Metformin and phenformin radiosensitize hypoxic tumor cells. CT26 and HCT116 tumor cells were treated with metformin or phenformin for 16 h at indicated concentrations. Afterward, cells were subjected to metabolic hypoxia and irradiated. The radiosensitivity of CT26 and HCT116 tumor cells under either aerobic or hypoxic conditions **(A)**. The radiosensitizing effect of phenformin **(B)** and metformin **(C)** under hypoxic conditions was assessed by colony formation assay. Data are shown from at least three experiments as mean ± SEM.

**FIGURE 4:**
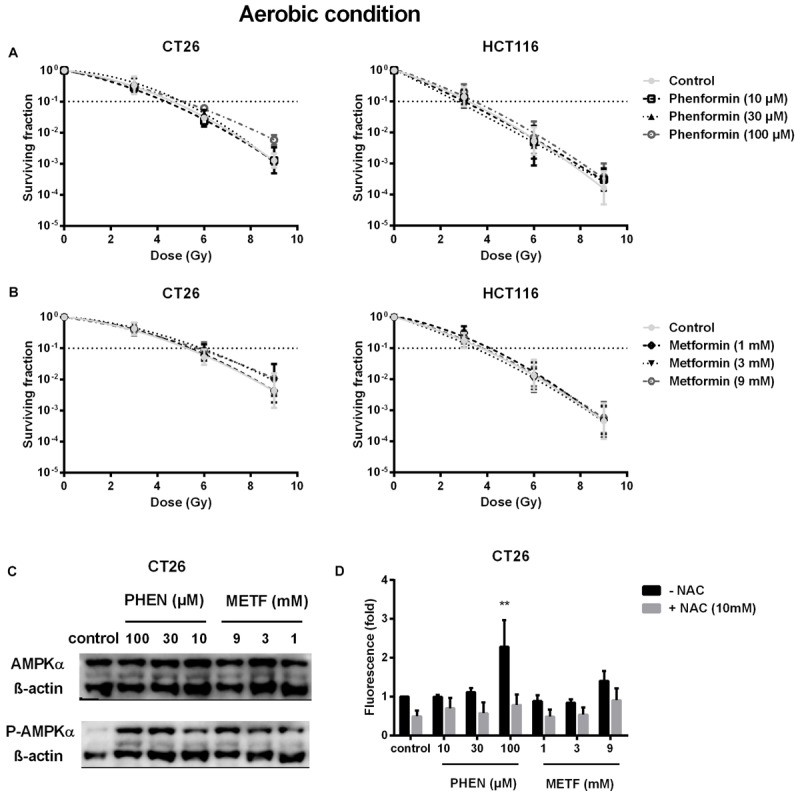
Metformin and phenformin are ineffective in enhancing intrinsic radiosensitivity of tumor cells although pAMPK and ROS production are upregulated. CT26 and HCT116 tumor cells were treated with metformin (METF) or phenformin (PHEN) for 16 h at indicated concentrations. The aerobic radiosensitivity of phenformin **(A)** and metformin **(B)** was assessed by colony formation assay. Phosphorylation of AMPK was examined by Western blotting **(C)**. NAC (10 mM) was added 1 h prior and during treatment. The intracellular level of ROS was detected by flow cytometry **(D)**. Data are shown from three experiments as mean ± SEM. Two-way ANOVA with Bonferroni’s multiple comparison test was used to calculate statistics: ^∗^*p* < 0.05, ^∗∗^*p* < 0.01, ^∗∗∗^*p* < 0.001, ^∗∗∗∗^*p* < 0.0001.

Decrease of OCR is a potent strategy to reduce tumor hypoxia and overcome hypoxia-induced radioresistance (Secomb et al., 1995; Diepart et al., 2012; Jiang et al., 2013; Lin and Maity, 2015; Zhou et al., 2016). We therefore asked whether metformin and phenformin could radiosensitize hypoxic tumor cells. Radiation experiments were performed in micropellets, a simplified metabolic hypoxia model used to prove the concept. Compared with normoxia, we indeed found a severely impaired radioresponse, with oxygen enhanced ratio of 2.29 and 2.45 for CT26 and HCT116 tumor cells, respectively (**Figure 3A**), indicating the existence of a deep hypoxia. In line with profound oxygen sparing (**Figures 2A,B**), phenformin overcame hypoxic radioresistance with enhancement ratios of 1.75 and 2.87 at 100 μM for CT26 and HCT116 tumor cells (**Figure 3B**). With respect to metformin, it improved the hypoxic radiosensitivity of CT26 and HCT116, respectively, by 1.72- and 2.86-fold at a dose of 9 mM, a 90 times higher concentration than phenformin (**Figure 3C**). In addition to tumor hypoxia, intrinsic radioresistance of tumor cells limits the efficacy of radiotherapy as well. Previously, both metformin and phenformin have shown to improve the intrinsic radiosensitivity of tumor cells through activation of the AMPK pathway (Wang et al., 2015a) and overproduction of ROS (Miskimins et al., 2014; Zhang et al., 2014). In our settings, although metformin and phenformin upregulated the phosphorylation of AMPK and induced the production of ROS, no enhanced intrinsic radiosensitivity could be detected under aerobic conditions (**Figures 4A–D**), suggesting that the intrinsic radiosensitizing effect of metformin and phenformin is cell line dependent.

### Metformin and Phenformin Enhanced Radioresponse of CT26 Tumors

**FIGURE 5:**
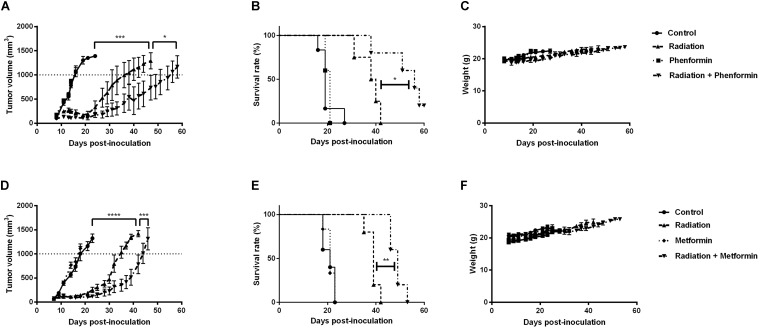
Metformin and phenformin enhance radioresponse of CT26 tumor. Metformin (300 mg/kg) and phenformin (200 mg/kg) were administered through oral gavage for 10 times to CT26 tumor-bearing mice, and radiation (9Gy) was delivered on the first day of treatment. Tumor growth delay in mice treated with radiation, phenformin or Metformin, and the combination **(A,D)**. Survival curves of mice euthanized when tumor diameter reached 15 mm **(B,E)**. Assessment of toxicity by body weight loss **(C,F)**. An ordinary one-way ANOVA with Tukey’s multiple comparisons test and a survival analysis was used to calculate statistics: ^∗^*p* < 0.05, ^∗∗^*p* < 0.01, ^∗∗∗^*p* < 0.001, ^∗∗∗∗^*p* < 0.0001.

The *in vitro* findings were further validated in CT26 tumor-bearing mice. Radiation alone at 9Gy delayed tumor growth by 17 days, measured at a tumor volume of 1000 mm^3^ (**Figures 5A,D**). Metformin (300 mg/kg) or phenformin (200 mg/kg) alone did not show any anti-tumor effect. While combined with radiation, both of them substantially delayed tumor growth by 26 and 40 days and enhanced radioresponse by 1.3- and 1.5-fold, respectively. As a result, the medium survival rate of tumor-bearing mice was significantly increased after treated with radiation in combination with metformin or phenformin (**Figures 5B,E**). Importantly, metformin or phenformin applied orally for 10 days was safe without inducing noticeable toxicity (**Figures 5C,F**). Altogether, in accordance with the *in vitro* findings, metformin and phenformin are effective in overcoming hypoxic radioresistance and improving radioresponse of colorectal tumor.

## Discussion

Preclinical evidence suggests that anti-diabetic drug metformin and phenformin can efficiently eliminate cancer cells and reduce metastases as a consequence of inhibition of mitochondrial complex I and activation of the AMPK signaling pathway. Next to the direct cytotoxic effect, our study demonstrates that metformin and phenformin are able to overcome hypoxia-induced radioresistance through inhibition of mitochondrial complex I, wherein phenformin exhibited a greater effect than metformin.

Complex I is a rate limiting step for electron transport chain in mitochondria, driving the synthesis of energy-carrying molecule ATP and in the meantime consumption of oxygen (Matsuzaki and Humphries, 2015). Inhibition of complex I as one of the anti-cancer mechanisms of metformin and phenformin has drawn quite some attention in the last several years (Andrzejewski et al., 2014; Miskimins et al., 2014; Boukalova et al., 2016; Velez et al., 2016). Whereas about two decades ago, this action of metformin has already been reported in hepatocytes, which contributes to the anti-diabetic as well as the side effects (El-Mir et al., 2000; Owen et al., 2000). In our study, we found that at a μM level, phenformin profoundly inhibited complex I activity in CRC cells with about 100-fold more potency than metformin (mM). This enormous difference between these two compounds is most likely due to the distinct physical properties. They share a similar chemical structure, however, phenformin is lipophilic and can readily permeate cells while metformin is hydrophilic, requiring an organic cation transporter (OCT) for cell entry. In normal conditions, OCTs are highly expressed in enterocytes in the intestine and hepatocytes in the liver, reflecting the pharmacokinetics of metformin that is absorbed in the intestine and then delivered to the liver to slow down glycogenesis (He and Wondisford, 2015). Besides normal tissue, OCTs are overexpressed in malignancies such as colorectal, prostate, and ovarian cancer (Segal et al., 2011; Tashiro et al., 2014; Obinata et al., 2016), and the downregulation of OCTs limits the antineoplastic effects of metformin (Segal et al., 2011). In our set up, the OCT expression although was not examined, it has been previously proved to be expressed in the applied cell lines (Kitada et al., 2008; Wheaton et al., 2014).

Clinically, the standard doses of metformin for diabetic patients are 1,500–2,500 mg per day with maximum plasma levels in the range of 10–25 μM (Graham et al., 2011). As aforementioned, metformin exerts its anti-cancer effects through multiple mechanisms, some of the actions are well attainable at μM levels, such as activation of AMPK via phosphorylation of Thr172 subunit (He and Wondisford, 2015). However, the concentrations required inhibiting complex I in our and other studies are much higher than the plasma levels (Wheaton et al., 2014), making people question the clinical relevance of these findings. Somehow, metformin at mM levels are likely achievable in clinic in CRC owing to the specific location and characteristics. Firstly, in intestine where CRC is located, at standard doses for diabetic patients, metformin could reach mM levels, which is most probably due to the high expression of OCTs in enterocytes (Bailey et al., 2008). Of note, efforts have been made to augment the activity of metformin; by attaching a lipophilic substituent, inhibitory effect of metformin toward complex I is potentiated about 1000-fold (Kalyanaraman et al., 2017). Secondly, it has been shown that cancer cells exhibiting higher mitochondrial respiratory activity are more sensitive to biguanide drugs (Hsu et al., 2015). In CRC, in contrast to the dogma that glycolysis is the predominant metabolism in cancer cells, the rate of oxygen consumption is higher in carcinomas as compared with normal adjacent tissues (Kaldma et al., 2014). This indicates that a reversed Warburg effect may exist in colorectal carcinomas, which may lead to a more predisposition of CRC cells to biguanides. Finally, OCTs and complex I have both reported to be higher expressed in the cancerous than the matched normal tissue in CRC (Yokoo et al., 2008; Wallace et al., 2016), suggesting a possibility of a selective cytotoxicity of metformin to CRC cells.

In agreement with the different extent of inhibitory activity toward complex I, phenformin displayed stronger cytotoxicity to CRC cells than metformin, in line with the observation from others in melanoma, breast, prostate, and lung cancer cells (Miskimins et al., 2014). However, at a concentration below 9 mM and 100 μM, respectively, for metformin and phenformin, for which were applied further to explore the radiosensitization, no cytotoxicity was observed. Seemingly, at these concentrations, inhibition of complex I caused decrease of ATP and increase of ROS, which normally lead to cytotoxicity, are compensated by upregulated glycolysis. Indeed, glucose deprivation or glycolysis inhibitors synergize the cytotoxic effect of metformin and phenformin (Wheaton et al., 2014).

Hypoxia, as a result of an imbalance between oxygen supply and consumption, is a common feature of solid tumors and a well-defined risk factor for radiotherapy failure (Gray et al., 1953). To date, numerous tools have been explored and developed to overcome hypoxia-induced radioresistance (Overgaard, 2007). However, their clinical application is hampered due to the practical limitations and toxicity. As an alternative approach, inhibition of oxygen consumption is proposed and demonstrated to be more efficient at alleviating hypoxia than increasing oxygen delivery (Secomb et al., 1995). Several drugs that target different components of the mitochondrial electron transport chain including complex I, II, III, and IV, indeed substantially counteracted hypoxic radioresistance via increased tumor oxygenation (Jordan et al., 2002; Crokart et al., 2007; Diepart et al., 2012; Jiang et al., 2013; Ashton et al., 2016). Glucocorticoids as an inhibitor of cytochrome c oxidase (complex IV) significantly promoted tumor oxygenation and enhanced the tumor radiosensitivity although administration of glucocorticoids somewhat decreased perfusion (Crokart et al., 2007). Insulin, as an inducer of nitric oxide that inhibits complex II, increased both tumor oxygenation and radioresponse in a liver and fibrocarcoma mouse tumors, with more tumor growth delay than carbogen breathing (95% O_2_, 5% CO_2_) (Jordan et al., 2002). Metformin is shown to reduce tumor hypoxia fraction and enhance radioresponse as well through inhibition of tumor cell respiration while little is known about phenformin (Zannella et al., 2013). In line, we found that metformin and phenformin as complex I inhibitors decreased oxygen consumption and enhanced hypoxic radiosensitivity of CRC cells by about 2-fold at doses of 9 mM and 100 μM, respectively. Recently, mitochondrial glycerophosphate dehydrogenase (MGPDH), a key enzyme connecting oxidative phosphorylation and glycolysis, is shown to be inhibited by metformin (Madiraju et al., 2014). Interestingly, this effect leads to reduction of mitochondrial respiration in thyroid cancer–derived cell lines (Thakur et al., 2018). As a next step, it could be important to explore whether MGPDH inhibition is attributable to the hypoxic radiosensitization of metformin and phenformin.

Next to hypoxic radiosenstization, both metformin and phenformin have been reported to radiosensitize aerobic tumor cells, including breast, lung, and pancreatic cancer cells (Storozhuk et al., 2013; Zhang et al., 2014; Wang et al., 2015a,b). The proposed mechanisms are inhibition of mTOR via activation of AMPK or upregulation of ROS production via disruption of antioxidant systems. mTOR inhibitors, such as rapamycin (Nagata et al., 2010), BEZ235 (Chang et al., 2014), and TAK228 (Miyahara et al., 2017), and pharmacological ROS insults, such as buthionine sulphoximine (Leung et al., 1993), auranofin (Wang et al., 2017), and piperlongumine (Matschke et al., 2016), overcame intrinsic radioresistance, leading to delayed tumor growth in different tumor models. Nevertheless, in the CRC cells used in our study, we found no evidence that metformin or phenformin affects intrinsic radiosensitivity. The intrinsic radiosensitivity of tumor cells is influenced by various factors, including DNA repair capacity, redox protein network, and expression patterns of oncogenes and tumor suppresses genes. The active extent of these factors differs in varying types of cancer, which might interpret the distinct response.

In concordance with the *in vitro* hypoxic radiosensitization, in syngeneic tumors, we found that metformin enhanced radioresponse and significantly increased the medium survival rate of tumor-bearing mice, which is in line with the findings in a CRC xenograft model that metformin was administered 30 min prior to radiotherapy (Zannella et al., 2013). In our study, the radiosensitizing effect was observed at a dose that is close to the maximal recommended safe dose via oral gavage (Song et al., 2012); in the other study, although it was 25% of the daily dose of patients, metformin was injected intravenously that could markedly boost plasma levels (Zannella et al., 2013). It is worth mentioning that different tumor models possess diverse characteristics, thus their susceptibility toward metformin and phenformin may differ. In spheroids of hypopharyngeal carcinoma cells and CRC cells, metformin and phenformin inhibited cellular respiration and reduced hypoxic fractions, but were ineffective in spheroids of lung cancer cells (Ashton et al., 2016). In addition, in a lung cancer xenograft model, although intraperitoneal injection of metformin and phenformin resulted in higher plasma concentrations than oral administration, the hypoxic fraction of the tumor was not affected by the treatments (Iversen et al., 2017). Whereas using the same lung cancer xenograft, phenformin improved radioresponse with a mechanism ascribed to activation of the AMPK pathway (Wang et al., 2015a). Hereby, our study is the first, to the best of our knowledge, to provide evidence that phenformin could radiosensitize colorectal tumor with a mechanism linked to a decrease of OCR via inhibition of complex I. We cannot exclude that other mechanisms might implicate in, such as boosting tumor immune surveillance. Metformin and phenformin are indeed known to inhibit the activity of pro-tumor MDSC and macrophages, and to enhance the generation of anti-tumor memory CD8+T-cell, consequently improving the efficacy of anti-cancer vaccine and PD-1 blockade (Eikawa et al., 2015; Kim et al., 2017; Scharping et al., 2017). Our preliminary data suggest that phenformin could deplete MDSC, while the effect of metformin and phenformin on memory CD8+T-cell is still under investigation.

Currently, dozens of clinical trials with metformin are initiated focusing mainly on breast and prostate cancer. About 10 on-going prospective phase II clinical trials are launched to explore whether metformin may improve therapy outcomes or lower CRC incidence in patients without diabetes. Among them, three address neoadjuvant metformin incorporation of radiotherapy in locally advanced rectal cancer (NCT02437656, NCT03053544). One is initiated in our institution, where metformin is combined with neoadjuvant chemoradiation to improve tumor radioresponse and patient outcome for locally advanced cT3-4 rectal cancer (EudraCT number: 2017-000814-50). Unlike metformin, the research wave of phenformin is just beginning with only one clinical trial initiated in which phenformin is combined with chemotherapeutic agents to treat metastatic melanoma (NCT03026517). Intriguingly, phenformin combined with non-pharmacological measures (rational nutrition, moderate physical activity and so forth) has been prescribed to breast and gastrointestinal cancer patients, aiming to rehabilitate the metabolism in 1970–1990 (Dilman et al., 1988; Berstein, 2010). After 10 years follow-up, the studies unveiled that this regimen is associated with improved relapse-free and total survival. The downside of these studies was the absence of proper randomization; nevertheless, they provide the first clinical evidence that phenformin may improve the outcome of cancer patients. Admittedly, phenformin has a predisposition to cause lactic acidosis in diabetic patients with renal failure, however, this side effect may be manageable by prescreening kidney function. Taken into account that the duration of phenformin administration in cancer patients would be shorter from its prior clinical use for diabetes, thus there is a possibility of a decreased incidence of severe side effects. In addition, it was reported that supplementation of glycolysis inhibitor with phenformin might avoid the risk of lactic acidosis and in the same time potentiate the antitumor effect (Lea et al., 2011; Gravel et al., 2014; Miskimins et al., 2014).

Taken together, in this study, we demonstrate that metformin and phenformin inhibit mitochondrial complex I and thereby block cellular respiration of CRC cells, leading to enhanced radioresponse. The radiosensitizing effect of metformin has been acknowledged with clinical trials initiated in combination with radiotherapy, while for phenformin it is still a completely blank page in clinical settings. Based on our and other’s findings, phenformin next to metformin merits clinical investigation as well and may represent an effective and inexpensive means to improve radiotherapy outcome.

## Ethics Statement

This study was carried out in accordance with the recommendations of the Ethical Committee for use of laboratory animals of the Vrije Universiteit Brussel. The protocol was approved by the Ethical Committee for use of laboratory animals of the Vrije Universiteit Brussel.

## Author Contributions

SdM and HJ conceived and designed the experiments, and wrote the manuscript. SdM and KL performed the experiments. CC and EB designed and helped performing the Seahorse experiments. TG helped with the radiation of the cells and mice. HW, ID, and VV revised the manuscript critically for important intellectual content. MDR and OF supervised the study and helped writing the manuscript. All authors discussed the results and contributed to the final manuscript.

## Conflict of Interest Statement

The authors declare that the research was conducted in the absence of any commercial or financial relationships that could be construed as a potential conflict of interest.
