# Supplementary material for: Antidiabetic Biguanides Radiosensitize Hypoxic Colorectal Cancer Cells Through a Decrease in Oxygen Consumption
Source: Front Pharmacol. 2018 Oct 3;9:1073. doi: 10.3389/fphar.2018.01073 (PMC6178882; doi:10.3389/fphar.2018.01073)
Supplement: Supplementary file 1 [file Table_1.docx]

Supplementary Material

Antidiabetic biguanides radiosensitize hypoxic colorectal cancer cells through a decrease in oxygen consumption

Sven de Mey^1^, Heng Jiang^1^, Cyril Corbet^2^, Hui Wang^1^, Inès Dufait^1,3^, Kalun Law^1^, Estelle Bastien^2^, Valeri Verovski^1^, Thierry Gevaert^1^, Olivier Feron^2^, and Mark De Ridder^1^*

^1^Department of Radiotherapy, Universitair Ziekenhuis Brussel, Vrije Universiteit Brussel, Brussels, Belgium.

^2^Pole of Pharmacology and Therapeutics (FATH), Institut de Recherche Expérimentale et Clinique (IREC), Université Catholique de Louvain, Brussels, Belgium.

^3^Laboratory of Molecular and Cellular Therapy, Vrije Universiteit Brussel, Brussels, Belgium.

**Correspondence:**Prof. Mark De Ridder
Laarbeeklaan 101, 1090 Brussels, Belgium
[mark.deridder@uzbrussel.be](mailto:mark.deridder@uzbrussel.be)
+3224776144

## Material and Methods

## 1.1 Mitochondrial complexes activity

After treatment, mitochondria were isolated from cancer cells and then seeded into a 96 well plate. Afterwards, the activity of mitochondrial complexes (I, II, III, and IV) was determined by Seahorse XF96 analyzer (Agilent, Belgium) as depicted in supplementary Fig. 1 and described elsewhere (Corbet et al. 2016). Briefly, the mitochondrial complexes activity is reflected by the change of oxygen consumption rate in different phases, following the injection of distinct compounds: 10mM pyruvate, 5mM malate, 2mM ADP, 1μM rotenone, 10mM succinate, 4μM antimycin A, and 10mM ascorbate plus 0.1mM TMPD.

## 1.2 MTT assay

Cytotoxicity of metformin and phenformin was assessed by MTT assay as described elsewhere (Wang et al. 2017). Briefly, after treatment, medium was aspirated and 50 μl MTT solution (5mg/ml) was added for 1.5h. Afterwards, 200 µl of MTT solvent (19:1 DMSO: HCL) was added and admixed to dissolve the formazan crystals generated inside of cells. Absorbance was measured at a wavelength of 540 nm by using a spectrophotometer (Bio-Rad, California, USA). Cell viability was determined by dividing the absorbance values of treated cells to that of untreated (control) cells.

## 1.3 Apoptotic assay

Apoptosis was analysed by flow cytometry using the double staining with lipophilic Annexin V and 7-amino actinomycin D (7-AAD) (Abcam, Cambridge, UK), as described elsewhere (Wang et al. 2017). Briefly, after treatment, cells were harvested and washed with FACS buffer. Thereafter, cells were resuspended in 100µl binding buffer (eBioscience) with 2.5µl Annexin V (eBioscience) and incubated for 20 minutes at room temperature. 7-AAD (5µl) was added five minutes before analysis. Early apoptotic cells (Annexin V-positive, 7-AAD-negative), necrotic/late apoptotic cells (double-positive), and living cells (double-negative) were determined by flow cytometry (BD LSR Fortsessa, BD Bioscience, Franklin Lakes, USA).

## Supplementary Figures


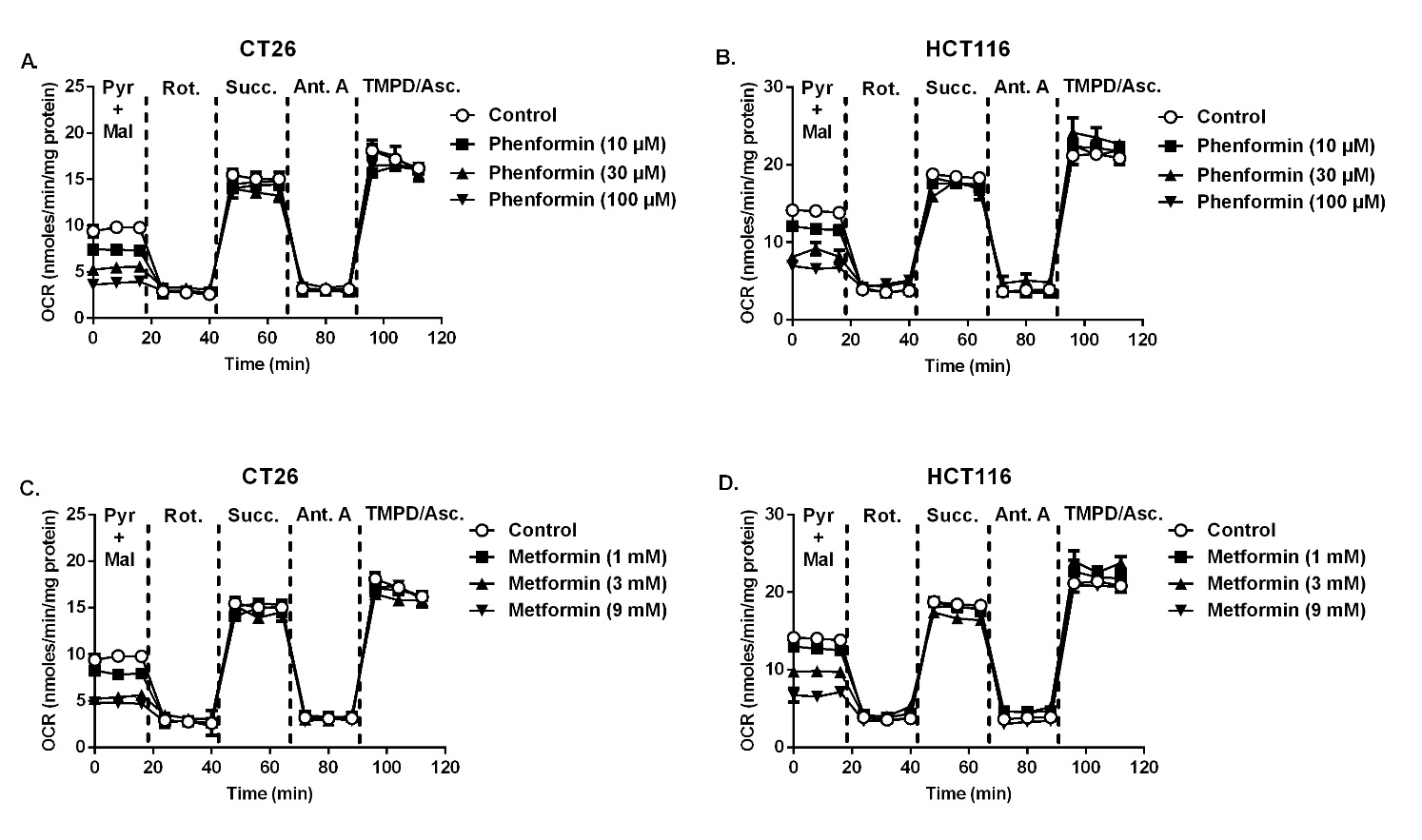


**Supplementary Figure 1.** **Representative curves of oxygen consumption rate (OCR) of isolated mitochondria.** After treatments, mitochondria were isolated from cells (CT26 (A,C) and HCT116 (B,D)) and then seeded into a 96 well plate. Afterwards, OCR was measured by using a Seahorse XF96 analyzer. To determine the mitochondrial complexes activity, reflected by the change of OCR in different phases, following compounds were injected: 10mM pyruvate, 5mM malate, and 2mM ADP (substrate for complex I), 1μM rotenone (complex I inhibitor), 10mM succinate (substrate for complex II), 4μM antimycin A (complex III inhibitor), 10mM ascorbate plus 0.1mM TMPD (substrate for complex IV). Oxygen consumption rate of each complex was calculated in the following way: complex I by subtracting OCR after addition of pyruvate and malate to that of rotenone, complex II by subtracting OCR after addition of succinate to that of rotenone, complex III by subtracting OCR after addition of succinate to that of antimycin A, and complex IV by subtracting OCR after addition of TMPD/ascorbate to that of antimycin A.


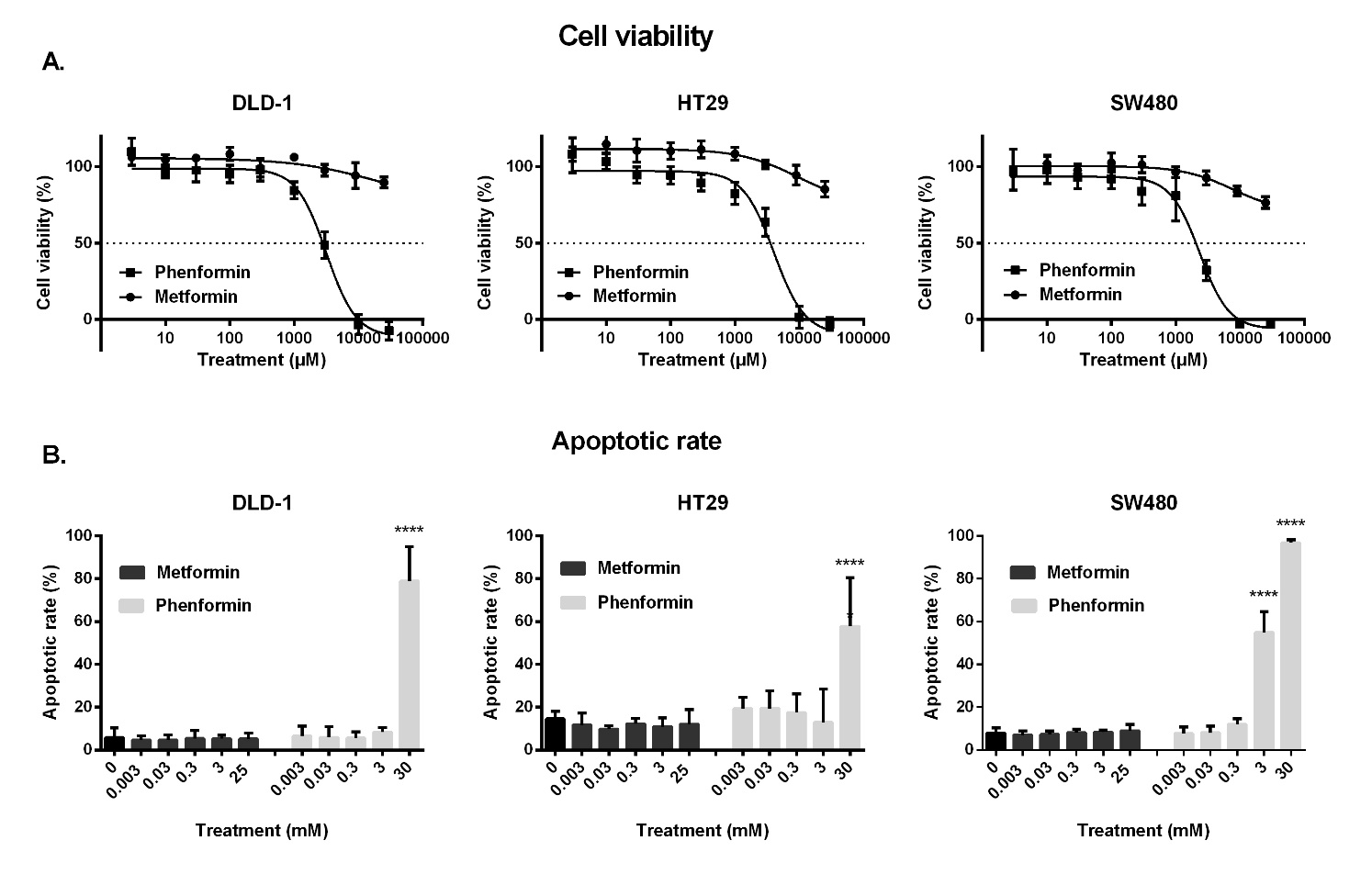


**Supplementary Figure 2: Cytotoxic effect of metformin and phenformin on CRC cell lines.** CRC cells (DLD-1, HT29 and SW480) were treated with metformin or phenformin with indicated concentrations for 16h. Cytotoxicity was determined by using MTT assay (A) and apoptosis (B) was assessed by flow cytometry using Annexin V/7-AAD staining. Data are shown from at least 3 experiments as mean ± SEM. One-way ANOVA with Dunnett’s multiple comparison test was used to calculate statistics: *p<0.05, **p<0.01, ***p<0.001, ****p<0,0001


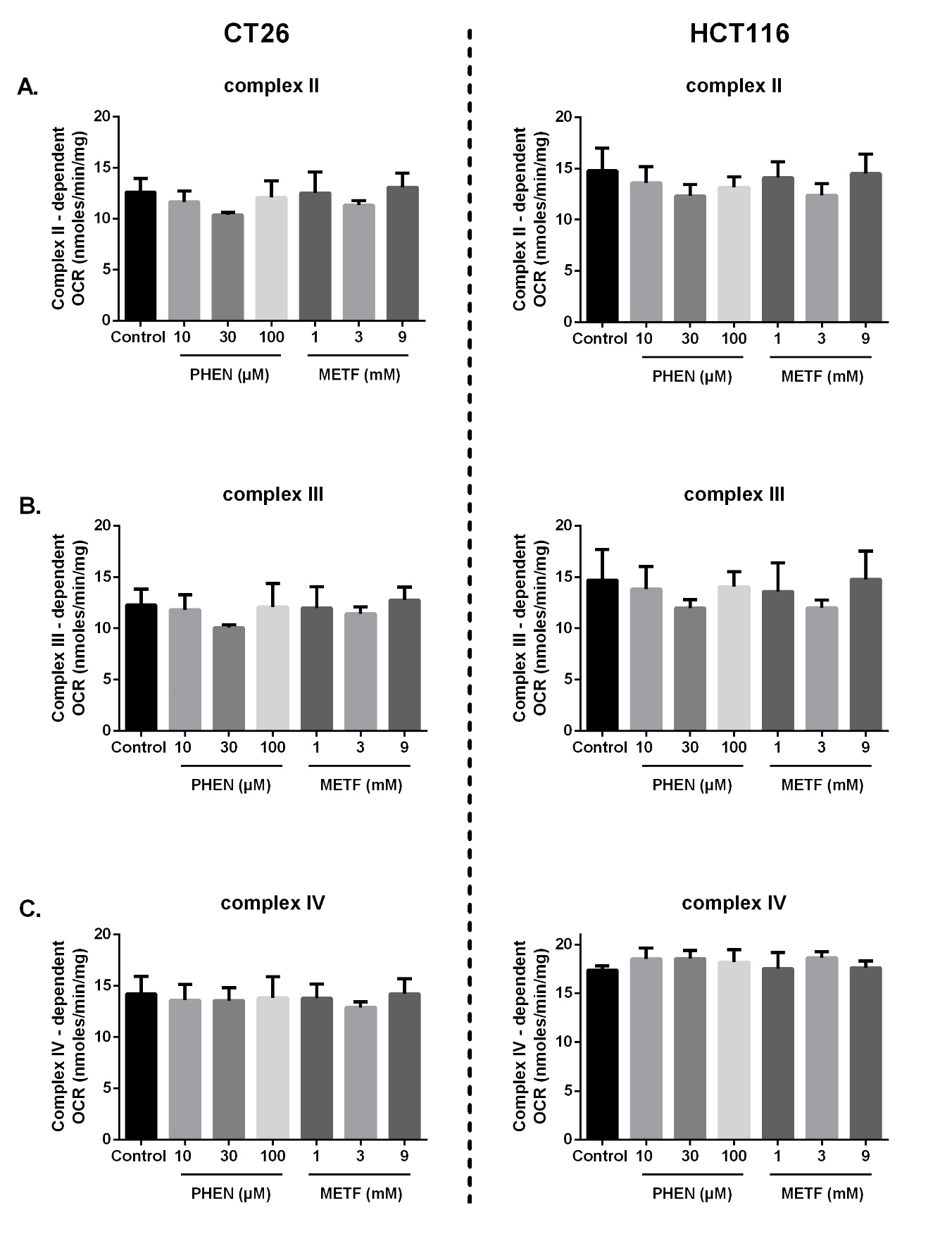


**Supplementary Figure 3: Metformin and phenformin do not inhibit complex II, III and IV activity.** CT26 and HCT116 tumor cells were treated with metformin (METF) or phenformin (PHEN) with indicated concentrations for 16h. Afterwards, mitochondria were isolated and activity of mitochondrial complexes (II, III and IV) was measured by using a Seahorse analyzer. Data is shown from 5 replicates as mean ± SEM. A one-way ANOVA with Dunnett’s multiple comparison test was used to calculate statistics: *p<0.05, **p<0.01, ***p<0.001, ****p<0,0001.

## References

Corbet, C., A. Pinto, R. Martherus, J. P. Santiago de Jesus, F. Polet, and O. Feron. 2016. "Acidosis Drives the Reprogramming of Fatty Acid Metabolism in Cancer Cells through Changes in Mitochondrial and Histone Acetylation." *Cell Metab* 24 (2):311-23. doi: 10.1016/j.cmet.2016.07.003.

Wang, H., S. Bouzakoura, S. de Mey, H. Jiang, K. Law, I. Dufait, C. Corbet, V. Verovski, T. Gevaert, O. Feron, D. Van den Berge, G. Storme, and M. De Ridder. 2017. "Auranofin radiosensitizes tumor cells through targeting thioredoxin reductase and resulting overproduction of reactive oxygen species." *Oncotarget* 8 (22):35728-35742. doi: 10.18632/oncotarget.16113.
